# A Case of Elephantiasis

**Published:** 1803-06-01

**Authors:** 

**Affiliations:** Manchester


### *A* Case *of* Elephantiasis; *communicated by Mr.* Ward, *of* Manchester.

THE success which attended the treatment of the following case leads me to suppose some benefit may result from its publication: I was reminded of it by a case somewhat similar, but of much longer standing, which fell under the care of Dr. Henning, of Zerbst. See vol. 9, p. 289.

Robert Walker, a native of Ireland, æt. 26, was admitted into the Infirmary, May 13, 1801, with a large tumour on the left leg, and a considerable, but more diffused swelling of the right. His account of the origin and progress of the disease was as follows.

Nine years ago he began to perceive an itching and cracking of the skin, accompanied by a watery discharge and swelling about the inner ancle of the left leg. The enlargement increasing, he became a patient in the hospital at Armagh, and afterwards in that at Tyrrol; and since his discharge, had been under the care of several eminent surgeons, but obtained no relief. He then came to England, and procured a recommendation to the Liverpool Infirmary; but not being taken in, he came to this place. Three years ago the disease began to shew itself in the right leg, attacking the same part, and commencing with the same symptoms as in the left, but the enlargement was trifling comparatively. About the same time he was attacked with rigors, pain in the head and back, *maziness*, and an irresistible propensity to sleep, which continued two or three days, and returned once a month or six weeks. He had had the itch two years before the first appearance of the complaint, which he thought had not not been properly treated, and which was the only probable cause he could assign.

The enormous size of the principal tumour, and a wish to examine it at my leisure, induced me to admit him, for I had no expectation of his receiving any benefit. It was moveable, firm, and elastic, and occupied nearly the whole of the space between the knee and ancle joints, commencing about an inch above the latter, where it measured 30 inches in circumference, and terminating about an inch and an half or two inches below the former, where I imagine it must have measured about 20. So abrupt was its termination, particularly at its lower part, and so equally was the limb surrounded by it, as to render its form not unlike that of a churn*. The skin, as far as the disease extended, had all the characteristic marks of Elephantiasis†: the sensibility was impaired; the joints of the toes were larger and the foot broader than natural: the man's face was not deformed, nor was his voice hoarse. His expression, however, was so rapid as to be often unintelligible; but this defect had subsisted, in a less degree, a long time. The right leg was not half so much enlarged as the left; in other respects the appearances were nearly the same. It is remarkable that he had walked nine miles the morning of his admission, without experiencing any inconvenience except what arose from the weight of these appendages.

An operation, all circumstances considered, did not seem advisable; I therefore resolved on trying the effect of pressure; the best mode of applying which appeared to be that adopted by Mr. Baynton, in the cure of ulcers.

The tumefied parts were well washed and rubbed with tepid water and soap two or three times a week, and every morning long narrow strips of adhesive plaster were applied tight, and a proper bandage over all. By this mode of treatment they were speedily and considerably reduced; and by persevering in it about five months, (when he was discharged for irregularity) both limbs were nearly restored to their natural shape and size.

During his stay in the house he was attacked pretty regularly once a month with nausea and pain of the stomach,

* I regret that it did not occur to me to have a drawing made from it till it was too late; but a tolerable correct idea may be formed from the above description.

† Cul. Nos. Med. V. ii. Cl. 3. O. 3. G. 87.

stomach, which gave way to an emetic, followed by a dose of rhubarb and calomel, and a tonic medicine.

Five months after his discharge, I received a letter from him, stating, that his limbs were in the same state as when he left the infirmary, and declaring his conviction that he should be as sound a man as any in Ireland, might he be permitted to come in again for another month.

*Manchester, May 12.*

